# Custom integration of a magnetic‐field monitoring system into a 32‐channel MRI head coil

**DOI:** 10.1002/mrm.30314

**Published:** 2024-09-29

**Authors:** Tim Schmidt, Yoojin Lee, Zoltan Nagy

**Affiliations:** ^1^ Laboratory for Social and Neural Systems Research (SNS Lab), University of Zurich Zurich Switzerland; ^2^ Institute for Biomedical Engineering, ETH Zurich and University of Zurich Zurich Switzerland

**Keywords:** head coil, industrial design, magnetic field monitoring, MRI

## Abstract

**Purpose:**

Customizing a Siemens 32‐channel coil for use in a Philips 3T MRI system with incorporated magnetic field probes for collecting high‐quality MRI and magnetic‐field monitoring data concurrently.

**Methods:**

The development process of the custom coil involved several (iterative) phases. Standard temporal SNR and B_1_^+^ data were collected with the 32‐channel Siemens and for reference the 32‐channel/8‐channel Philips head coils before and after the custom coil was made compatible with the 3T Philips Achieva system, and magnetic field probes were installed into it along with ancillary electronics around it. Quality assurance tests were conducted in each of the build phases to ensure that the modifications did not affect MRI or field‐monitoring data negatively. To test the finished custom coil, we collected high angular resolution diffusion imaging (HARDI) datasets on a spherical silicon oil phantom both with and without concurrent field monitoring and a 32‐channel Philips coil without concurrent field monitoring, where the latter two served as reference scans to assess the improved performance of the custom coil with field monitoring. Similar HARDI‐MRI data were also collected in vivo with the finished custom coil together with field monitoring data.

**Results:**

The custom coil provided excellent temporal SNR especially at the edges where cortical gray matter is expected. When using concurrent field monitoring in HARDI acquisitions, the custom coil alleviated ghosting artifacts in phantom data and provided in vivo images with 1.4‐mm isotropic resolution.

**Conclusion:**

The custom MRI coil with integrated magnetic‐field monitoring probes provided improved imaging performance.

## 1 INTRODUCTION

Magnetic‐field monitoring^1^, ^2^ is a method for tracking the spatio‐temporal magnetic field evolution with a set of magnetic field probes^3^ during the standard operation of an MRI scanner. For obvious reasons, the magnetic field probes (henceforth *probes*) cannot be positioned at the actual locations (e.g., inside the brain) where we want knowledge about the field dynamics. Instead, the probes are arranged outside the imaging volume of interest, and to these data spherical harmonic components of various orders are fit.^4^

One challenge for such a setup is arranging the probes without impairing standard operating procedures or affecting data quality of the MRI scanner negatively, while providing high‐quality data from the probes. To this end, various solutions have been proposed. For example, the probes can be positioned either inside the inner surface^5^, ^6^ or outside the outer surface^7^, ^8^ of the plastic covering of the head coil–both of which have some limitations. When the probes are positioned inside the head coil, they further restrict the volume that is reserved for the head, cushioning, and headphones, and necessitate the routing of additional cabling out of the head coil near the human participant. On the other hand, placing them outside runs the inherent problem of making field measurements farther from the imaging volume of interest. For completeness, it is also possible to position the probes within the imaging volume of interest,^2^ but in this case concurrent monitoring of in vivo experiments is not possible.

To simultaneously overcome or alleviate all these mentioned limitations, one may place the probes inside the plastic housing of the head coil, which avoids encroaching on the space within the coil, keeps the probes closer to the imaging volume of interest, and allows concurrent monitoring of in vivo experiments. Challenges also exist with this solution. For example, the spatial positioning of the constellation of probes is more restricted, which may limit the quality of the spherical harmonic fits. Furthermore, the proximity of the coil and probe hardware may result in unwanted electromagnetic interactions that could disturb either the probe signal, the coil signal, or both. Nevertheless, previous efforts have succeeded with installing the probes in this manner,^9^, ^10^, ^11^ and this is the procedure that the present article follows. Among these, the commercially available coil has only 16 receive channels and is specialized for imaging the brain with a small inner diameter.

We present a custom installation of 16 probes and ancillary electronics into and around a modified 32‐channel receive‐only head coil. The final product was built around a large number of criteria (Table 1) to arrive at an aesthetically pleasing, safe, and ergonomic coil that produces high‐quality signal for both MRI and magnetic‐field monitoring.

**TABLE 1:** Design criteria.

Ergonomics and patient comfort
A single operator must be able to (un)install the custom coil
The inside surface of the coil that accommodates the volunteer's head must remain unmodified
Line of sight could not be blocked
Durability
Minimal cable length from probes through RF traps to Tx/Rx front‐end box avoiding kinking/tearing
All parts must be 3D printable with durable plastic
Standard MRI operating procedures
3D‐printed from MR‐invisible plastic
The Tx/Rx box was kept as close and as low as possible to preserve the line of sight to back‐projection screen
Design must remain compatible with eye tracking during functional MRI experiments
Design must remain compatible with the manufacturer's front‐view and back‐view mirror system
An aesthetically pleasing final design to avoid increasing anxiety of volunteer participants
Safety
No sharp edges
Magnetic field probes most not have direct contact to volunteer's skin
Final design must not affect local emergency procedures negatively
Technical aspects
Siemens coil (flat base) must be compatible with Philips patient table (curved surface)
The center of the inner volume of the coil must be as close as possible to isocenter of the magnet
The field probes are distributed in such a way that the dynamic fields can be measured up to the third order of the spherical harmonic expansion.
Miscellaneous
Price and time constraints

Abbreviations: Rx, receive; Tx, transmit.

## 2 METHODS

The ultimate aim of the project was to develop a custom 32‐channel MRI coil for use in a 3T Achieva scanner (Philips Healthcare, Best, The Netherlands) with incorporated magnetic field probes. Apart from the custom coil, henceforth called S32ch (“S” for the SNS Lab), the vendor's 32‐channel and 8‐channel coils, henceforth referred to as V32ch and V8ch, were also included as reference.

### 2.1 Building the S32ch custom coil

Several sets of boundary conditions, whose optimums often opposed each other, had to be considered to arrive at the final design. These boundary conditions included safety, durability, ergonomics, as well as high‐quality performance from the MRI coil, the field‐monitoring setup, and the image‐reconstruction pipeline. For example, for a well‐conditioned estimation of the spherical harmonic fields from the 16‐point measurements,^1^, ^4^ the probes should be distributed in space in an irregular fashion (e.g., not in a line or on plane). Simultaneously, each of the probes should be well away from active and passive electronic components of the MRI coil as well as from sharp edges of the plastic housing. To reach an optimal compromise in line with such, sometimes opposing, criteria was only possible through a carefully considered initial design followed by a large number of iterative steps that included testing and modifying the setup.

**FIGURE 1:**
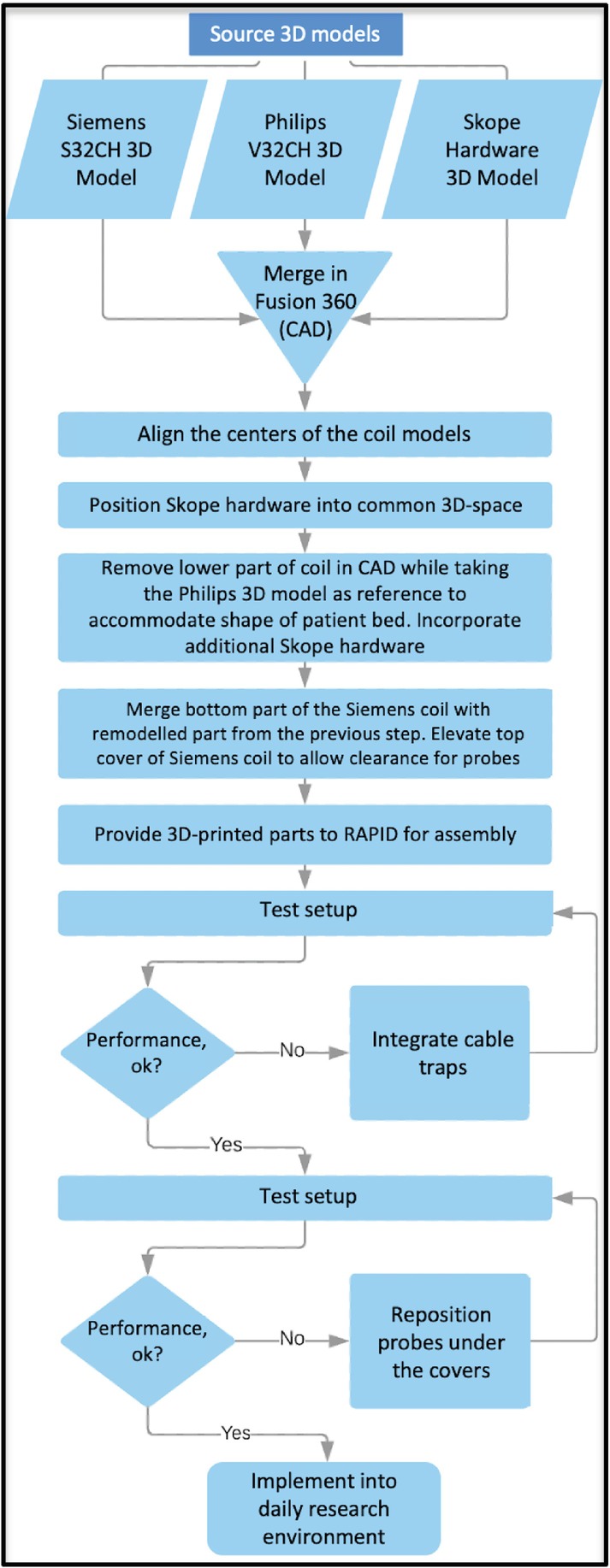
Flowchart illustrating the iterative process between design and engineering steps. S32CH, SNS Lab 32‐channel; V32CH, vendor's 32‐channel.

More generally, these iterations were contained in several phases. Throughout these phases, both the MRI coil and the magnetic‐field monitoring setup were tested, and the industrial design aspects of the coil housing were refined as needed. Although it is not possible to clearly separate the industrial design from all other aspects, a flowchart in Figure 1 provides a helpful, albeit simplified, overview.

To assess the progress of the customization, data (described in detail later) were collected to calculate temporal SNR (tSNR) and the transmit RF field (B_1_^+^) in each of the phases. To assess the performance of the magnetic‐field monitoring setup, the FID lifetime of the probes was measured and/or images were reconstructed offline from the raw data of both the S32ch MRI coil and the 16 probes.

The various phases of the coil customization will be described next.

#### 2.1.1 Phase 1

We sourced a second‐hand Siemens 3T 32ch coil in the fall of 2017, and before any modification, we used it in a 3T Trio scanner in March 2018 (Siemens Healthineers, Erlangen, Germany) to assess its base performance. Using the V32ch and V8ch coils, tSNR and B_1_^+^ data were also collected in our 3T Achieva scanner. An additional B_1_^+^ map was also acquired with another V8ch coil that included 16 probes.^5^ Finally, the FID lifetimes of the 16 field‐monitoring probes were measured on their own before installation, while lying near the isocenter of the 3T Achieva scanner.

#### 2.1.2 Phase 2

The S32ch coil was made compatible with the 3T Philips Achieva platform by Rapid Biomedical (Rimpar, Germany). Before further modification for installing the field monitoring hardware, the coil was shipped back and tSNR and B_1_^+^ data were collected in our 3T Philips Achieva scanner.

#### 2.1.3 Phase 3

To install the magnetic‐field probes and necessary ancillary electronics and to make the Siemens coil compatible with the Philips scanner, a large portion of the plastic housing had to be redesigned. The new design accommodates the curved surface of the Philips patient bed, hides all 16 probes and their cabling under the outer covers, and carries the transmit (Tx)/receive (Rx) front‐end of the probes. In several iterations, various constellations of the probe positions were tested, and the expected noise propagation performance was calculated for the different constellations from Eq. (8) in Barmet et al.^1^

The best‐performing constellation required a probe to be installed between the eye holes on the coil and as caudal as possible. For this, the plastic covering of the anterior part of the coil had to be elevated.

#### 2.1.4 Phase 4

After installing the probes into the newly designed housing, MRI data quality worsened, the tSNR values were drastically reduced, and the B_1_^+^ maps were severely distorted, necessitating the installation of RF cable traps around the probe cables. In the hopes of simplifying the final design, at first all 16 cables were fed through a few cable traps, but data quality could only be recovered fully if each of the 16 probes received a dedicated RF trap. This necessitated a redesign of the coil housing again, creating a gap between the coil proper and the Tx/Rx front‐end. The 16 cable traps were installed in a scaffold with three rows in a staggered “wine bottle holder” style. Additional data were collected again on our Philips Achieva scanner for tSNR and B_1_^+^ maps. The final testing of the finished custom coil was performed by RAPID in December 2019.

### 2.2 Quality assurance tests during the design and building process

Given that the excellent neuroimaging performance of the original 32ch Siemens coil was the primary motivation for the development of this custom solution, it was of paramount importance that its performance was maintained despite all the modifications to the coil itself and the installation of the additional magnetic‐field monitoring equipment in its direct proximity. Three outcome measures of imaging performance were used. First, tSNR was calculated from a time series of gradient‐echo EPI^12^ data from a gel phantom—similar to that in the FBIRN protocol.^13^ The initial test of the 32ch Siemens coil in Phase 1 was performed in a 3T Trio scanner with voxel size = 3.0 × 3.0 × 2.5 mm^3^, slice gap = 0.5 mm, and TR/TE = 3430/30 ms. All other scans on the 3T Philips Achieva scanner were acquired with voxel size = 3.0 × 3.0 × 3.0 mm^3^ and TR/TE = 2800/30 ms. The approximately 10‐min time series was first rigid body–aligned to the first EPI volume, and the slow voxel‐wise signal trend was removed with a second‐order polynomial fit. Subsequently, the tSNR was calculated voxel‐wise as the ratio of the temporal mean and temporal SD. The second outcome measure, a B_1_^+^ field map was obtained with a dual TR method that was developed by Yarnykh^14^, ^15^ and further optimized locally.^16^ Relevant acquisition parameters were TR1/TR2 = 16/138 ms, TE = 2.2 ms, and nominal flip angle = 60°.

### 2.3 Field‐monitored imaging with the finished S32ch coil

As the final outcome measure, we collected high angular resolution diffusion imaging (HARDI)^17^ data on a silicon‐oil phantom with an EPI sequence that was locally programmed and included a number of modifications: (a) synchronize the scanner and skope system (b) trigger the skope system for acquiring the field monitoring data (c) turn off certain corrections that the vendor does to the raw k‐space data (d) several other practical modifications (e.g. keeping shimming constant). Relevant imaging parameters included: 84 slices, one b_0_ image and 64 diffusion directions (b = 1000 s/mm^2^ and b = 2000 s/mm^2^) that were evenly distributed over a hemisphere, 1.4‐mm isotropic resolution, volume TR = 16 801 ms, TE = 90/103 ms for b = 1000 s/mm^2^ and b = 2000 s/mm^2^, respectively, sensitivity encoding (SENSE) acceleration factor of 3,^18^ 200 × 200 × 117 mm^3^ field‐of‐view, 90° flip angle and an EPI^12^ readout that resulted in an 18:47‐min scan for each b‐value. The magnetic field dynamics were concurrently monitored during the EPI readout using 16 NMR probes integrated into the head coil and a skope acquisition system (Skope Magnetic Resonance Technologies AG, Zurich, Switzerland). Before the HARDI scan we carried out a low‐resolution bipolar (3.0 × 3.0 × 1.4 mm^3^) multiple gradient‐echo sequence (five echoes, first TE = 4.6 ms, and ΔTE = 1.15 ms) to estimate a B_0_ field map using all gradient echoes. The B_0_ field–mapping data and the monitored EPI trajectories were later used in offline image reconstruction to attenuate image artifacts and distortions and reduce both static and dynamic field disturbances.^4^ Furthermore, we also reconstructed the images without the B_0_ mapping data to qualitatively compare it with the vendor‐reconstructed images.

For the offline reconstruction of the images, an in‐house reconstruction implementation was used, the algorithms of which are described in more detail in Bertram et al.^4^

After demodulation of the probes phases and removing up to second‐order concomitant field contributions,^1^, ^19^ a second‐order spatial spherical harmonics field model was fit to the measured field dynamics to account for long‐term eddy currents resulting from the strong diffusion gradients.^20^ The B_0_ acquisition was also monitored and images corrected with a second‐order spherical harmonics expansion. We achieved the best image quality when using a second‐order spatial model, where we have more probes than spherical basis terms and are therefore less prone to overfitting. Representative reconstructed slices with a first‐order and third‐order spatial spherical harmonics field model to the probe phase data are shown in Figure S1.

For comparison, we also acquired diffusion‐weighted MRI scans without concurrent field monitoring using both the S32ch and V32ch coils. The reconstructed images were obtained directly from the scanner without any additional postprocessing. In these scans, we omitted slice‐wise phase navigators (which are usually used to counter the effects of the drifting scanner frequency), because in the phantom we did not plan to combine the different diffusion directions to fit a model in each voxel.

Finally, 1 healthy male participant was scanned in agreement with our local ethics guidelines using the S32ch coil with concurrent field monitoring and the acquisition protocol described previously. A tensor was fit to these data with *dtifit* in FSL (version 6.0.7.10; http://fsl.fmrib.ox.ac.uk/fsl), from which color‐coded images were generated that represent the direction of the eigenvector (V1) corresponding to the largest eigenvalue and modulated by the local fractional anisotropy.

## 3 RESULTS

**FIGURE 2:**
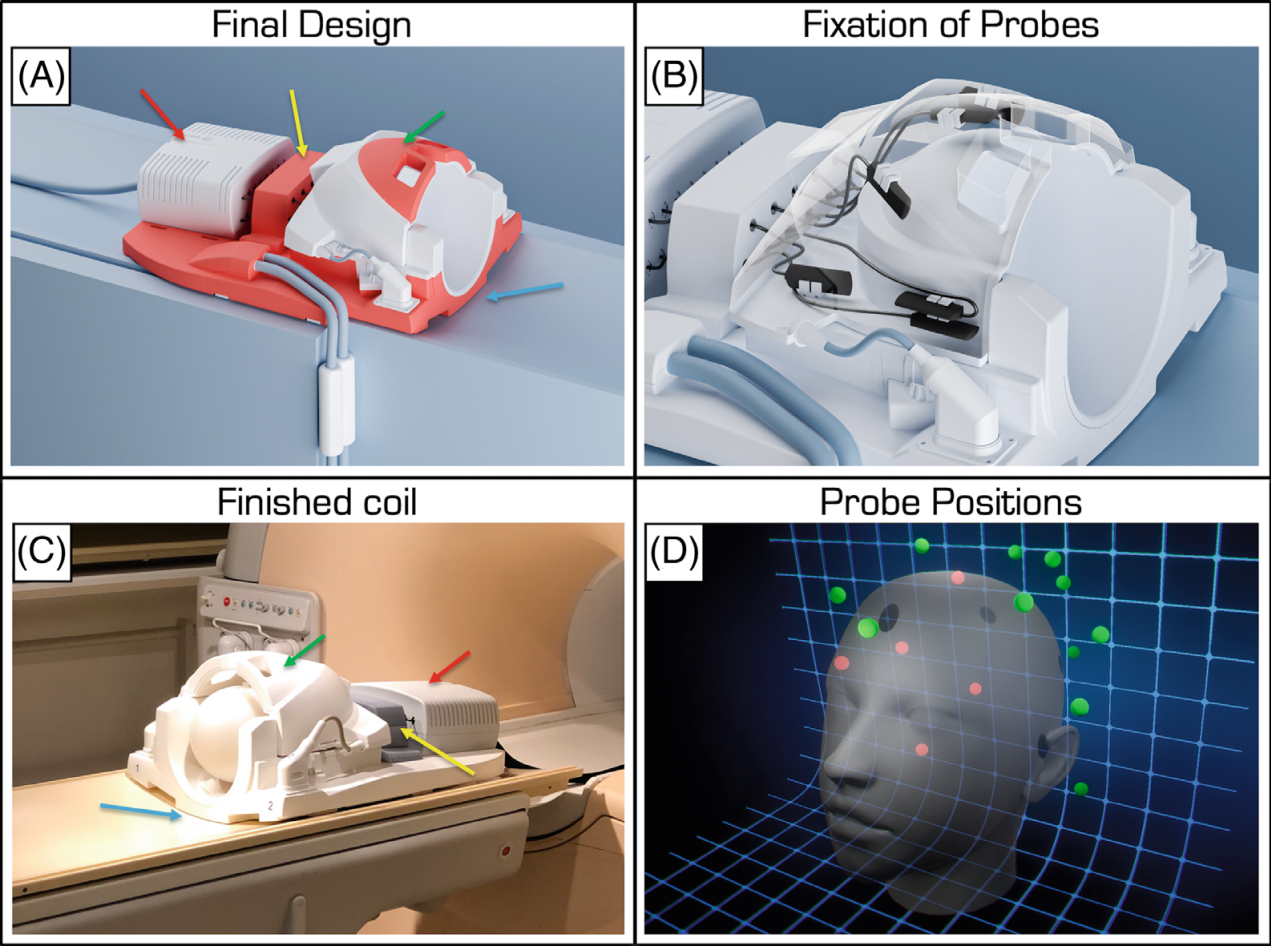
The final installation conformed to a large set of criteria (Table 1). For example, the design had to match the curved table top of the 3T Achieva Phillips scanner (*blue arrow*), provide a platform for the transmit (Tx)/receive (Rx) front‐end of the field‐monitoring setup (*red arrow*), house the RF cable traps (*yellow arrow*), accommodate the field‐monitoring probes under the plastic covers (*green arrow*), minimize cable lengths from probes to the Tx/Rx front‐end box and feed conveniently through the RF traps, and ensure coil handling by a single operator. (A) Rendering of the final design with the (re)designed parts shown in red. (B) Mock‐up illustration of approximate probe positions under the plastic outer covers of the coil and the methods of securely fixing each probe in place. The small white clamp of each probe was glued to the inner surface of the coil housing with MR‐invisible glue. The probes fit tightly into these clips but were further secured by cable ties (not shown). (C) Photograph of the finished and working coil. (D) Rendering of probe positions in 3D space. The human head model is for illustration only. The red spheres would be blocked out by the head from this camera angle.

Figure 2 displays the final design, which contains all components of the coil itself, the magnetic‐field monitoring setup, as well as the additional RF cable traps, built to satisfy the various boundary conditions that are detailed Table 1 and the figure legend.

**FIGURE 3:**
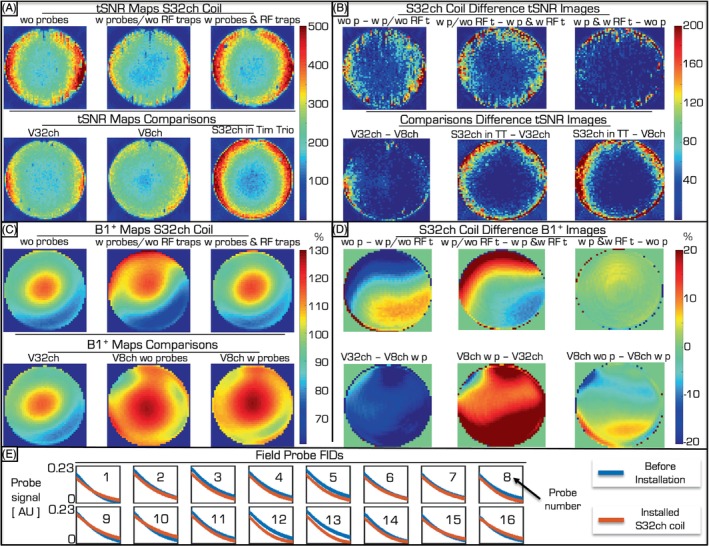
Quality control data from the various build and design phases of the S32ch coil. (A) The top row provides tSNR maps of a gel phantom in the S32ch coil, measured in the Achieva scanner before the probes were installed (*left*), after the probes were installed but before RF cable traps were added (*middle*), and the finished coil (*right*). The second row provides comparative data from the V32ch coil (*left*), the V8ch coil (*middle*) from an Achieva scanner, as well as the S32ch coil used in a Tim Trio scanner before any modification to it. (B) Various pair‐wise comparisons of the tSNR maps, where w p = “with probes,” wo p = “without probes,” w RF t = “without RF traps,” wo RF t = “without RF traps,” and TT = “Tim Trio.” Note that the final tSNR of the S32ch coil was similar to that of the original coil while plugged into its native platform. (C) The top row provides B_1_^+^ maps of the S32ch coil, measured in the Achieva scanner before the probes were installed (*left*), after the probes were installed but before RF cable traps were added (*middle*), and the finished coil (*right*). The second row provides comparative data from the V32ch coil (*left*), the V8ch coil without field probes (*middle*), and another V8ch coil with field probe installation—all of them measured in the Achieva scanner. Note that adding RF cable traps to the S32ch setup was necessary to avoid distorting the B_1_^+^ field by the probes and their cabling. However, the V8ch coil performed comparably without RF cable traps before and after adding the field‐monitoring probes. (D) Various pairwise comparisons of the B_1_^+^ maps. Note that the final B_1_^+^ map of the S32ch coil was similar to that before the probes were installed. (E) FIDs from the individual probes before and after installation into the S32ch coil.

Figure 3A,B provide the tSNR and B_1_^+^ maps, respectively, from different phases of the modification process. The tSNR of the S32ch coil was well above that of the V8ch coil. The S32ch coil also provided higher tSNR (especially at the edges where brain gray matter is expected) than the V32ch coil, which could be expected given the latter has a larger diameter. Importantly, the tSNR produced by the final S32ch coil was nearly that of the coil before any modification. The B_1_^+^ maps in Figure 3C reinforce that neither any modification to the coil itself nor the installation of the probes and necessary cabling caused major distortions in the transmit RF field. Notably, in contrast with an 8ch installation,^5^ cable traps were necessary to ensure this.

FID lifetimes of the probes were hardly affected by the installation (Figure 3E). This ensures that the setup is suitable for monitoring the spatio‐temporal magnetic field dynamics during experiments with long readout times (e.g., high resolution EPI or spiral acquisitions).

**FIGURE 4:**
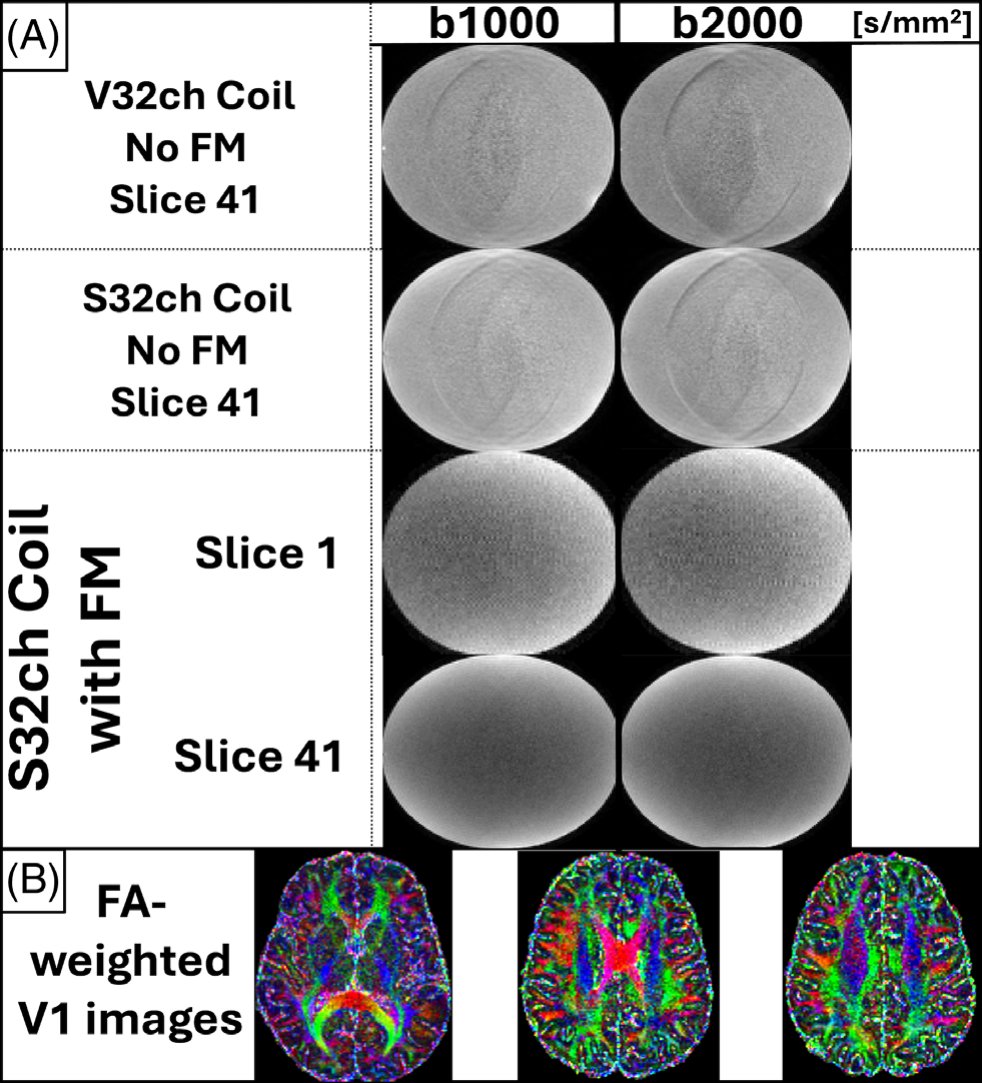
(A) Silicon phantom images of the HARDI scans obtained using both the V32ch coil without field monitoring (No FM) and the finished S32ch coil with and without concurrent field monitoring. These scans cover b‐values of 1000 s/mm^2^ (*left column*) and 2000 s/mm^2^ (*right column*) for the diffusion direction [−0.55, 0.31, 0.77]. The final data quality with field monitoring is shown from two slices that cover the middle and edge of the imaging volume, to illustrate that data quality is equally good, even farther away from the isocenter, where magnetic fields and field gradients are expected to deviate the most from their nominal value. Note that for all slices, the intensity units are windowed between 0 and 200. The images of the monitored data were slightly rescaled to ensure similar signal intensities. (B) Three axial slices from the monitored in vivo scan using the S32ch coil. Each slice displays the eigenvector (V1) corresponding to the largest eigenvalue of the diffusion tensor, modulated by the local fractional anisotropy (FA) (green, anterior–posterior; red, left–right; and blue, cranial–caudal directions.

Figure 4A displays representative slices from the diffusion‐weighted phantom scans with both the V32ch and S32ch coils—the latter both with and without concurrent field monitoring—and using diffusion weighting of b = 1000 s/mm^2^ and b = 2000 s/mm^2^. In the absence of field monitoring, both coils suffer from ghosting artifacts. Incorporating concurrent field monitoring yields substantial improvements in image quality. Remaining ghosting artifacts may have been caused by local eddy currents induced by gradient fields on nearby conductors, leading to a distorted field measurement. However, such residual ghosts were not discernible in the raw in vivo data (not shown), nor in the resulting color‐coded V1 images weighted by fractional anisotropy (Figure 4B).

In Figure S2, we show Slices 1 and 41 with 1000 and 2000 s/mm^2^ diffusion weighting (top and bottom row, respectively), reconstructed offline using the monitored trajectories but without the B_0_ mapping data.

## 4 DISCUSSION

We described the steps and considerations toward developing a custom coil that can concurrently collect MRI and magnetic‐field sensing data. The final version of the S32ch coil can be used by a single operator like other product coils and collects high‐quality, non‐diffusion data even when magnetic‐field monitoring is not required (Figure 3). Image artifacts in diffusion experiments can be mitigated with additional field monitoring (Figure 4). The project itself took approximately 2.5 years. Building another, similar coil would require considerably less time.

It would not be feasible to describe every aspect of the design and build processes. As such, neither the flowchart in Figure 1 nor Table 1 are complete but were meant to serve the reader in appreciating the complexity of the project. There were numerous additional small modifications, long discussions, and various aspects that were ultimately decided against and discarded or reversed. Nevertheless, the present article is meant to demonstrate that building such a hybrid coil is possible and manageable. Most of the human in vivo neuroimaging literature is based on data that were collected with the product coils that the scanner vendor provided or commercially available third‐party coils. A smaller fraction of the work relies on home‐built coils.^9^, ^11^ Drastically modifying a product coil, as presented here, is much less common. One principal aim of the present manuscript is to illustrate that, albeit challenging, a middle ground is possible between purchasing product coils and building one from scratch. Even those sites that do not have a dedicated RF lab can develop a specialized coil for their specific use case. This is analogous to buying a commercial car versus customizing a rally car vs building a Formula 1 car.

Although previous work has shown that image reconstructions, while correcting for up to third‐order spherical harmonic fields can be superior in diffusion imaging,^20^ we found correcting only for up to second order produced higher quality images. Estimating all 16 spherical harmonic terms of the first three orders with high fidelity with 16 measurements (i.e., the 16‐field monitoring probes installed in the S32ch coil) is challenging, and others have also opted to use the 16 measurements to estimate only nine unknowns of the first two orders.^21^ We left it for future work to identify the root cause and decide whether to solve it at the design stage or correct for it in postprocessing. Various methods have been put forth for the latter, postprocessing approach to recover the quality of third‐order reconstructions. For example, Dubovan et al. pointed out that measurements with probes farther from the isocenter can result in overfitting the higher‐order terms and suggest a step‐wise fitting approach, while Nussbaum et al. used an additional measurement, with which the probe signals themselves are corrected before fitting.^22^, ^23^

The principal motivation for embarking on this project was to perform cortical parcellation, based on high‐quality and high‐resolution HARDI data.^24^ As such, we welcomed the increased SNR of S32ch coil in the vicinity of brain cortical gray matter and were less concerned about receive nonuniformity. In cases in which a more uniform SNR or a larger coil is needed, the V32ch coil provides a useful alternative. Its larger diameter allows for a simpler installation, where the probes could be attached to the inner surface of the head enclosure—similar to the method by Kennedy et al. for the V8ch coil.^5^ The V32ch coil possesses the added benefit of having a dedicated hole at the cranial apex, which was originally meant for EEG cables, but can serve the same purpose for guiding the probe cables out of the coil.

The in vivo scan used acquisition parameters to purposely push the SNR limits. Despite the 1.4‐mm isotropic resolution, we acquired reasonable data in less than 19 min. Future effort will assess how much data quality could be improved with longer acquisitions (either increasing angular resolution or repeating and averaging the same diffusion directions) with spiral readouts^10^ in more modern scanners with stronger gradients—all of which would result in substantially improved SNR. Should the need arise for even higher resolution data, our experience suggests that an approximate 50‐ms EPI readout would be possible to monitor (Figure S3).

## 5 CONCLUSION

We have presented the steps for a custom integration of a set of magnetic field probes into a 32ch receive‐only head coil and made it compatible with a Philips 3T Achieva MRI system. The finished S32ch coil provided higher tSNR than other 32‐channel and 8‐channel coils and improved image quality in diffusion‐weighted scans with concurrent field monitoring and offline reconstruction. We presented the process of building a custom coil because alternatives are scarce:

- Buy the 3T head coil from skope
- Instead of concurrent field monitoring, reconstruct images on premonitored field trajectories
- Attach the clip‐on probes to the outside of the head coil
- Build your own coil from scratch

However, these alternatives do not cover all use cases nor are they available to all MRI laboratories.

## CONFLICT OF INTEREST

Nothing to report.

## Supporting information


**Figure S1.** The top row shows two representative offline reconstructed slices (80 and 41) acquired with the same diffusion direction [−0.55, 0.31, 0.77] and a b‐value of 1000 s/mm^2^. The measured field dynamics were fit to a first‐order spatial spherical harmonic field. Bottom row shows the same kind of images but with a third‐order spatial spherical harmonics field.
**Figure S2.** Top row provides Slices 1 and 41 of the diffusion‐weighted phantom scans with b = 1000 s/mm^2^, reconstructed offline without the B_0_ map but including the monitored trajectories fitted up to second spatial spherical harmonics order. Bottom row shows the same kind of images but with b = 2000 s/mm^2^ diffusion weighting.
**Figure S3.** Individual magnitude probe dynamics during the EPI readout (here, 37 ms long) as shown on the skope acquisition system. The path of signal loss suggests that an approximately 50‐ms readout would still be possible. Note that on the y‐axis are arbitrary intensity units.

## Data Availability

The CAD files include proprietary information from the various vendors and therefore cannot be shared publicly. However, the authors are willing to share any/all CAD files with those who obtain permission from the relevant vendors.
