# Supplementary material for: Custom integration of a magnetic‐field monitoring system into a 32‐channel MRI head coil
Source: Magn Reson Med. 2024 Sep 29;93(2):889–98. doi: 10.1002/mrm.30314 (PMC11604842; doi:10.1002/mrm.30314)
Supplement: Supplementary file 1 — Figure S1. The top row shows two representative offline reconstructed slices (80 and 41) acquired with the same diffusion direction [−0.55, 0.31, 0.77] and a b‐value of 1000 s/mm2. The measured field dynamics were fit to a first‐order spatial spherical harmonic field. Bottom row shows the same kind of images but with a third‐order spatial spherical harmonics field. Figure S2. Top row provides Slices 1 and 41 of the diffusion‐weighted phantom scans with b = 1000 s/mm2, reconstructed offline without the B0 map but including the monitored trajectories fitted up to second spatial spherical harmonics order. Bottom row shows the same kind of images but with b = 2000 s/mm2 diffusion weighting. Figure S3. Individual magnitude probe dynamics during the EPI readout (here, 37 ms long) as shown on the skope acquisition system. The path of signal loss suggests that an approximately 50‐ms readout would still be possible. Note that on the y‐axis are arbitrary intensity units. [file MRM-93-889-s001.pdf]

Supplementary Figures

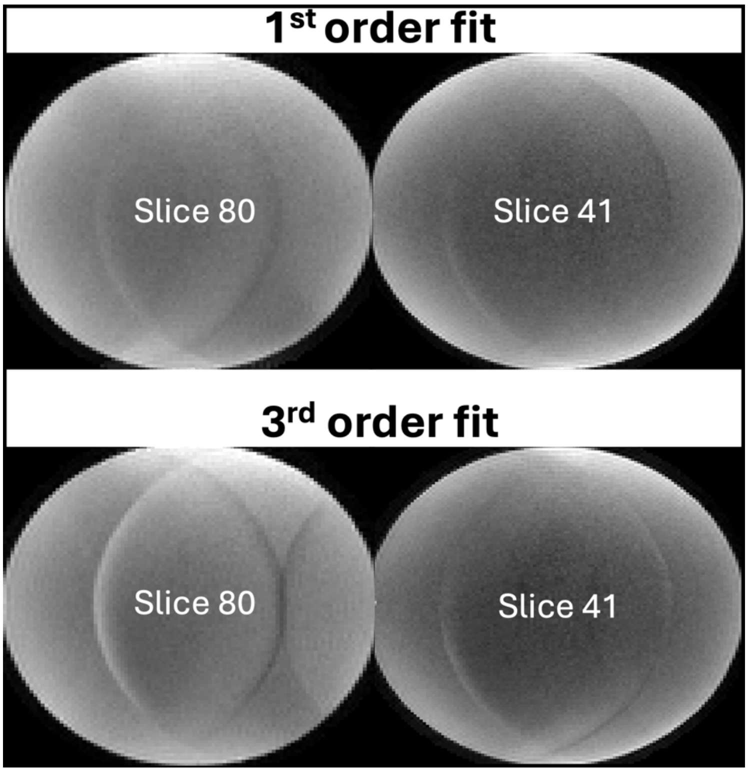

Figure S1. The top row shows two representative offline reconstructed slices (80 and 41) acquired with the same diffusion direction [-0.55, 0.31, 0.77] and a b-value of 1000 s/mm2. The measured field dynamics were fit to a 1st order spatial spherical harmonic field. Bottom row shows same kind of images but with a 3rd order spatial spherical harmonics field.

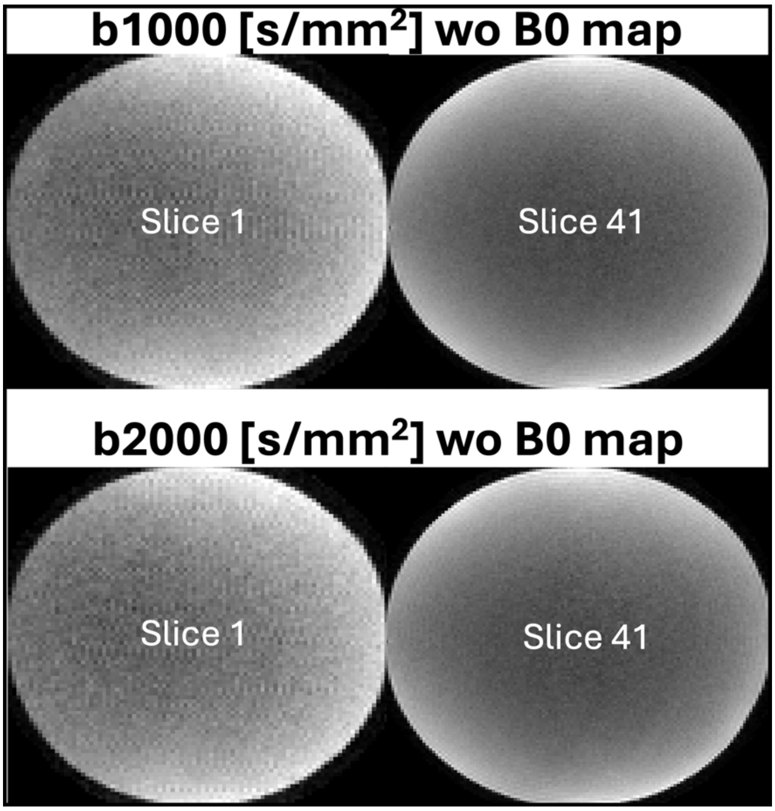

Figure S2. Top row provides slices 1 and 41 of the diffusion weighted phantom scans with b = 1000 s/mm^2, reconstructed offline without the B0 map but including the monitored trajectories fitted up to 2nd spatial spherical harmonics order. Bottom row shows the same kind of images but with b = 2000 s/mm^2 diffusion weighting.

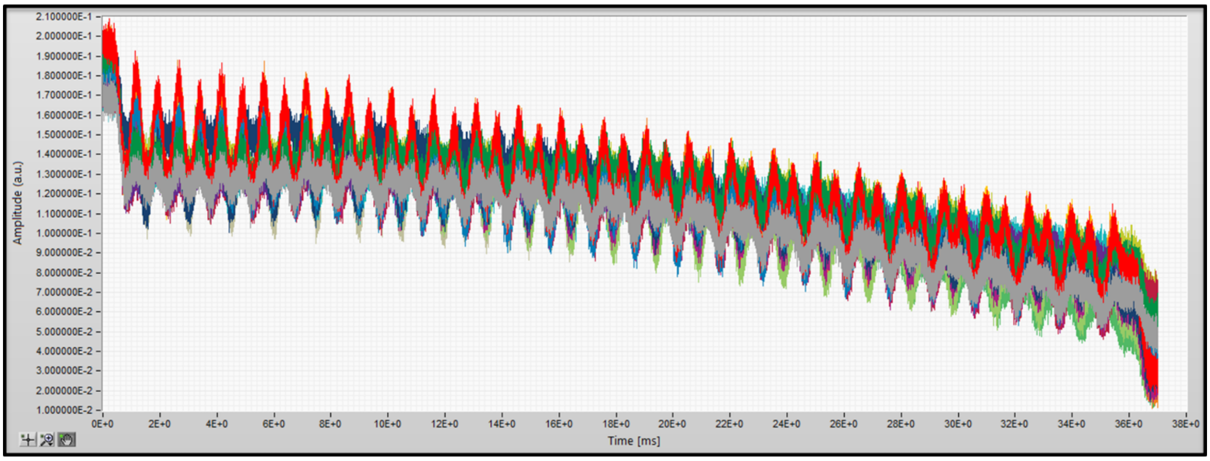

Figure S3. Individual magnitude probe dynamics during the EPI readout (here 37ms long) as shown on the skope acquisition system. The path of signal loss suggests that an approximately 50-ms readout would still be possible. Note that on the y-axis are arbitrary intensity units.
